# Global Critical Care: Add Essentials to the Roadmap

**DOI:** 10.5334/aogh.2546

**Published:** 2019-07-04

**Authors:** Carl Otto Schell, Abigail Beane, Raphael Kazidule Kayambankadzanja, Karima Khalid, Rashan Haniffa, Tim Baker

**Affiliations:** 1 Global Health—Health Systems & Policy, Department of Public Health Sciences, Karolinska Institutet, Stockholm, SE; 2 Centre for Clinical Research Sörmland, Uppsala University, Uppsala, SE; 3 Mahidol Oxford Tropical Research Unit, Bangkok, TH; 4 Network for Improving Critical care Systems and Training, Colombo, LK; 5 University of Malawi, College of Medicine, Blantyre, MW; 6 Department of Anaesthesia and Critical care, Muhimbili University of Health and Allied Sciences, Dar es Salaam, TZ; 7 Bloomsbury Institute for Intensive Care Medicine, Division of Medicine, University College London, London, UK

To the Editor,

We thank Diaz and co-authors for their recent review of Global Critical Care [1]. We agree that many health systems under-perform as they fail to prioritize illness severity and the care of the critically unwell, focusing instead on diagnoses and medical specialities. We also agree on the great potential for epidemiological, clinical, and implementation research in global critical care to improve patient outcomes. We would like to make one correction and suggest important additions to the roadmap.

1. Unfortunately, the review misrepresents a finding from our Vital Signs Directed Therapy (VSDT) study [2]. We did not conclude that “nursing-based vital signs–directed clinical response protocols may not improve outcomes” as claimed. In fact, although overall mortality rates were unchanged following the implementation of VSDT in an intensive care unit (ICU) in Tanzania, there was a 25% absolute reduction of mortality among hypotensive patients. Our conclusion after analysing process measures was that “a vital signs directed therapy protocol improved the acute treatment of abnormal vital signs in an ICU in a low-income country”. It is our belief that protocol-based instruments and task-sharing have great potential to improve critical care.
2. Improved recognition of critical illness and the delivery of basic care for critically ill patients throughout the hospital should be a central aspect of a global critical care roadmap. We have recently proposed the concept of Essential Emergency and Critical Care (EECC) – the care that all critically ill patients should receive in all hospitals in the world [3]. If the effective coverage of EECC was higher than today, many critically ill patients could be stabilized and survive without the need for care in an ICU, leading to improved outcomes at low cost. Well-equipped ICU beds could then be spared for the most unwell patients.
3. When assessing the global burden of critical illness, we agree that there are major limitations of extrapolating data from ICU admissions and incomplete diagnosis-based registries and that novel methodologies are needed. Further examples to those mentioned in the review include our ongoing multi-national Critical Illness Prevalence and Outcome Study (CRISPOS), which uses point-prevalence estimations, whereby all in-patients are assessed for critical illness. Similarly, as electronic health information systems start to proliferate in low- and middle-income countries, routinely collected hospital-wide data could be exploited [4].

We agree with the authors about the importance of cost-effectiveness. To maximise this, innovative tools that aim at getting the most out of the basics should be paramount in the roadmap for the future. Funders and researchers could reduce the risk that their investments in critical care end up benefitting just a few individuals by acknowledging that the quality of essential services *for all* is the foundation of critical care systems.
